# Supplementary material for: Evidence supported by Mendelian randomization: impact on inflammatory factors in knee osteoarthritis
Source: Front Med (Lausanne). 2024 May 28;11:1382836. doi: 10.3389/fmed.2024.1382836 (PMC11165061; doi:10.3389/fmed.2024.1382836)
Supplement: Supplementary file 1 [file Data_Sheet_1.ZIP › data/GCST90274792_5e6/792.leaveoneout.pdf]

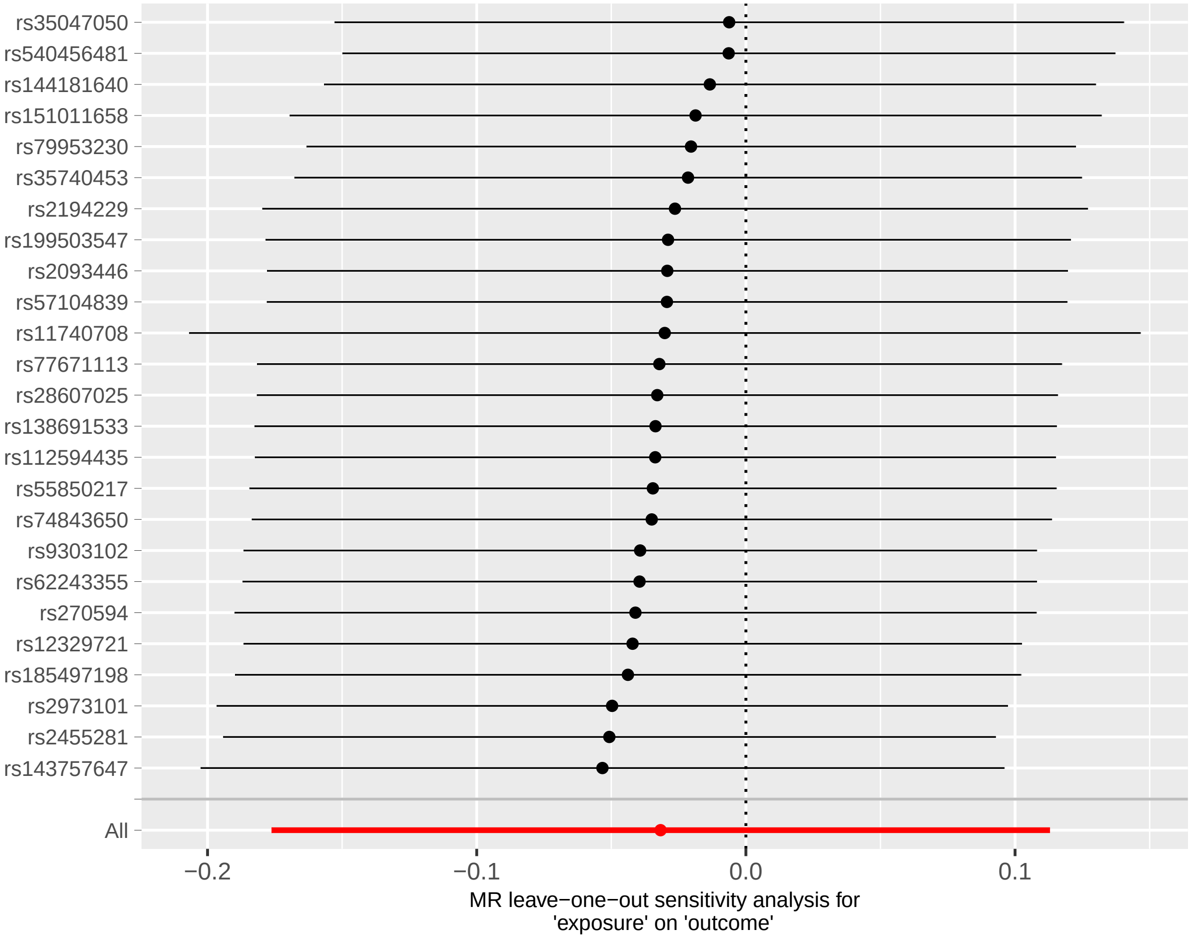
rs35047050
rs540456481
rs144181640
rs151011658
rs79953230
rs35740453
rs2194229
rs199503547
rs2093446
rs57104839
rs11740708
rs77671113
rs28607025
rs138691533
rs112594435
rs55850217
rs74843650
rs9303102
rs62243355
rs270594
rs12329721
rs185497198
rs2973101
rs2455281
rs143757647
All
−0.2
−0.1
0.0
0.1
MR leave−one−out sensitivity analysis for
'exposure' on 'outcome'
